# Fumarate hydratase is a critical metabolic regulator of hematopoietic stem cell functions

**DOI:** 10.1084/jem.20161087

**Published:** 2017-03-06

**Authors:** Amelie V. Guitart, Theano I. Panagopoulou, Arnaud Villacreces, Milica Vukovic, Catarina Sepulveda, Lewis Allen, Roderick N. Carter, Louie N. van de Lagemaat, Marcos Morgan, Peter Giles, Zuzanna Sas, Marta Vila Gonzalez, Hannah Lawson, Jasmin Paris, Joy Edwards-Hicks, Katrin Schaak, Chithra Subramani, Deniz Gezer, Alejandro Armesilla-Diaz, Jimi Wills, Aaron Easterbrook, David Coman, Chi Wai Eric So, Donal O’Carroll, Douglas Vernimmen, Neil P. Rodrigues, Patrick J. Pollard, Nicholas M. Morton, Andrew Finch, Kamil R. Kranc

**Affiliations:** 1 Medical Research Council Centre for Regenerative Medicine, University of Edinburgh, Edinburgh EH8 9YL, Scotland, UK; 2 Centre for Cardiovascular Science, Queen’s Medical Research Institute, University of Edinburgh, Edinburgh EH8 9YL, Scotland, UK; 3 Edinburgh Cancer Research UK Centre, Medical Research Council Institute of Genetics and Molecular Medicine, University of Edinburgh, Edinburgh EH8 9YL, Scotland, UK; 4 The Roslin Institute, University of Edinburgh, Edinburgh EH8 9YL, Scotland, UK; 5 Wales Gene Park and Wales Cancer Research Centre, Division of Cancer and Genetics, School of Medicine, Cardiff University, Cardiff CF10 3XQ, Wales, UK; 6 The European Cancer Stem Cell Research Institute, School of Biosciences, Cardiff University, Cardiff CF10 3XQ, Wales, UK; 7 Mater Children’s Private Hospital Brisbane, South Brisbane, Queensland 4101, Australia; 8 Department of Metabolic Medicine, The Lady Cilento Children’s Hospital, South Brisbane, Queensland 4101, Australia; 9 Department of Haematological Medicine, Division of Cancer Studies, King’s College London, London WC2R 2LS, England, UK

## Abstract

Strict regulation of stem cell metabolism is essential for tissue functions and tumor suppression. In this study, we investigated the role of fumarate hydratase (Fh1), a key component of the mitochondrial tricarboxylic acid (TCA) cycle and cytosolic fumarate metabolism, in normal and leukemic hematopoiesis. Hematopoiesis-specific *Fh1* deletion (resulting in endogenous fumarate accumulation and a genetic TCA cycle block reflected by decreased maximal mitochondrial respiration) caused lethal fetal liver hematopoietic defects and hematopoietic stem cell (HSC) failure. Reexpression of extramitochondrial Fh1 (which normalized fumarate levels but not maximal mitochondrial respiration) rescued these phenotypes, indicating the causal role of cellular fumarate accumulation. However, HSCs lacking mitochondrial Fh1 (which had normal fumarate levels but defective maximal mitochondrial respiration) failed to self-renew and displayed lymphoid differentiation defects. In contrast, leukemia-initiating cells lacking mitochondrial Fh1 efficiently propagated *Meis1*/*Hoxa9*-driven leukemia. Thus, we identify novel roles for fumarate metabolism in HSC maintenance and hematopoietic differentiation and reveal a differential requirement for mitochondrial Fh1 in normal hematopoiesis and leukemia propagation.

## Abstract (precis)

Guitart et al. performed an in vivo genetic dissection of the Krebs cycle enzyme fumarate hydratase (Fh1) in the hematopoietic system. Their investigations revealed multifaceted functions of Fh1 in the regulation of hematopoietic stem cell biology and leukemic transformation.

## Introduction

Successful clinical application of hematopoietic stem cells (HSCs) is critically dependent on their ability to give long-term multilineage hematopoietic reconstitution (Weissman and Shizuru, 2008). Multiple studies have revealed the paradigmatic transcription factors driving HSC self-renewal and differentiation to sustain multilineage hematopoiesis (Göttgens, 2015). Emerging evidence indicates that strict control of HSC metabolism is also essential for their life-long functions (Suda et al., 2011; Manesia et al., 2015), but the key metabolic regulators that ensure stem cell integrity remain elusive. Although highly proliferative fetal liver (FL) HSCs use oxygen-dependent pathways for energy generation (Suda et al., 2011; Manesia et al., 2015), adult HSCs are known to suppress the flux of glycolytic metabolites into the mitochondrial tricarboxylic acid (TCA) cycle and heavily rely on glycolysis to maintain their quiescent state (Simsek et al., 2010; Takubo et al., 2013; Wang et al., 2014). Whereas pharmacological inhibition of glycolytic flux into the TCA cycle enhances HSC activity upon transplantation (Takubo et al., 2013), severe block of glycolysis (i.e., *Ldha* deletion) and a consequent elevated mitochondrial respiration abolishes HSC maintenance (Wang et al., 2014). The switch from glycolysis to mitochondrial oxidative metabolism is essential for adult HSC differentiation rather than maintenance of their self-renewing pool (Yu et al., 2013). Leukemia-initiating cells (LICs) are even more dependent on glycolysis than normal HSCs (Wang et al., 2014). Partial or severe block in glycolysis (elicited by deletion of *Pkm2* or *Ldha*, respectively) and a metabolic shift to mitochondrial respiration efficiently suppress the development and maintenance of LICs (Lagadinou et al., 2013; Wang et al., 2014). Thus, the maintenance of adult self-renewing HSCs and LICs appears to depend critically on glycolysis rather than the mitochondrial TCA, which is thought to be less important for this process. However, thus far, the requirement for any of the TCA enzymes in FL and adult HSC and LIC maintenance has not been investigated.

Genetic evidence in humans indicates that rare recessive mutations in the *FH* gene encoding a TCA enzyme fumarate hydratase (Fh1) result in severe developmental abnormalities, including hematopoietic defects (Bourgeron et al., 1994). Consistent with this, we also found that monozygous twins with recessive *FH* mutations (Tregoning et al., 2013) display leukopenia and neutropenia (Table S1), thus suggesting a role for *FH* in the regulation of hematopoiesis. Mitochondrial and cytosolic fumarate hydratase enzyme isoforms, both encoded by the same gene (called *FH* in humans and *Fh1* in mice; Stein et al., 1994; Sass et al., 2001), catalyze hydration of fumarate to malate. Whereas mitochondrial Fh1 is an integral part of the TCA cycle, cytosolic Fh1 metabolizes fumarate generated during arginine synthesis, the urea cycle, and the purine nucleotide cycle in the cytoplasm (Yang et al., 2013). Autosomal dominant mutations in *FH* are associated with hereditary leiomyomatosis and renal cell cancer, indicating that *FH* functions as a tumor suppressor (Launonen et al., 2001; Tomlinson et al., 2002). Given that *FH* mutations have been associated with hematopoietic abnormalities and tumor formation, here, we investigated the role of *Fh1* in normal and malignant hematopoiesis.

## Results

### *Fh1* is required for FL hematopoiesis

**Figure 1.**
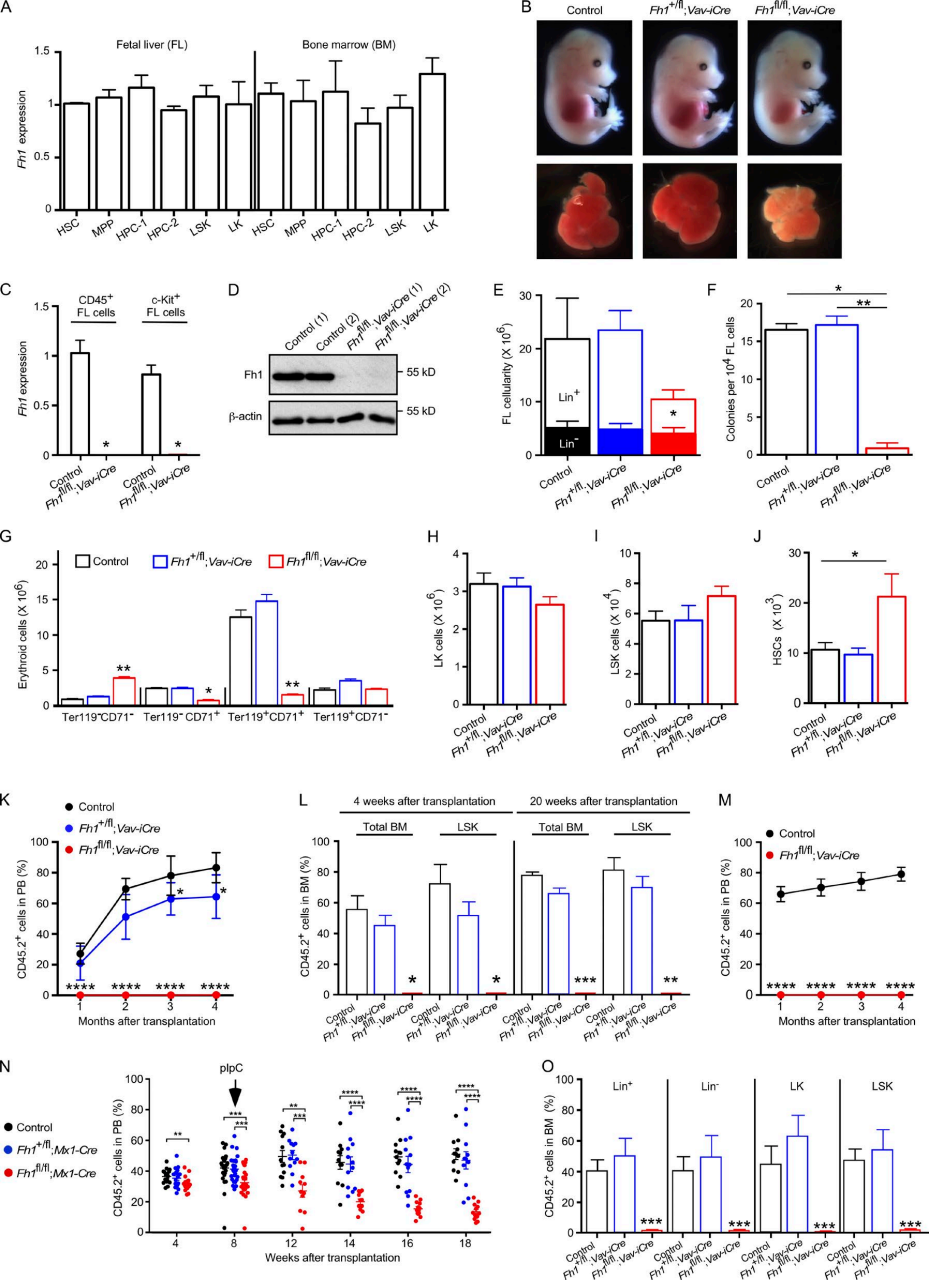
**Hematopoiesis-specific *Fh1* deletion results in severe hematopoietic defects and loss of HSC activity.** (A) Relative levels of *Fh1* mRNA (normalized to *Actb*) in HSCs, multipotent progenitors (MPP), HPC-1 and HPC-2 populations, and LSK and LK cells sorted from 14.5-dpc FLs and BM of C57BL/6 adult (8–10 wk old) mice. *n* = 3. (B) FLs from 14.5-dpc *Fh1^fl/fl^;Vav-iCre* embryos are smaller and paler compared with *Fh1^+/fl^;Vav-iCre* and control embryos. (C) The absence of *Fh1* transcripts in *Fh1^fl/fl^;Vav-iCre* FL CD45^+^ and c-Kit^+^ cells. Control, *n* = 3; *Fh1^fl/fl^;Vav-iCre*, *n* = 6. (D) Western blots for Fh1 and β-actin in FL c-Kit^+^ cells. (E) Total cellularity (the sum of Lin^+^ and Lin^−^ cell numbers) in 14.5-dpc FLs of the indicated genotypes. Control, *n* = 17; *Fh1^+/fl^;Vav-iCre*, *n* = 11; *Fh1^fl/fl^;Vav-iCre*, *n* = 9. (F) CFC assay with FL cells. Control, *n* = 11; *Fh1^+/fl^;Vav-iCre*, *n* = 8; *Fh1^fl/fl^;Vav-iCre*, *n* = 4. (G) Erythropoiesis in 14.5-dpc FLs. Data are arranged from least to most differentiated: Ter119^−^CD71^−^, Ter119^–^CD71^+^, Ter119^+^CD71^+^, and Ter119^+^CD71^–^. Control, *n* = 11; *Fh1^+/fl^;Vav-iCre*, *n* = 8; *Fh1^fl/fl^;Vav-iCre*, *n* = 4. (H–J) Total number of LK cells (H), LSK cells (I), and HSCs (J) in 14.5-dpc FLs. Control, *n* = 17; *Fh1^+/fl^;Vav-iCre*, *n* = 11; *Fh1^fl/fl^;Vav-iCre*, *n* = 9. (K and L) Percentage of donor-derived CD45.2^+^ cells in PB (K) and total BM and the BM LSK cell compartment (L) of the recipient mice transplanted with 100 FL HSCs. *n* = 5–8 recipients per genotype. At least three donors were used per genotype. (M) Percentage of CD45.2^+^ cells in PB after transplantation of 200,000 total FL cells. *n* = 3–4 recipients per genotype. At least three donors were used per genotype. (N and O) Acute deletion of *Fh1* from the adult hematopoietic system. 5 × 10^5^ unfractionated CD45.2^+^ BM cells from untreated *Fh1^fl/fl^* (control), *Fh1^+/fl^;Mx1-Cre*, and *Fh1^fl/fl^;Mx1-Cre* C57BL/6 (8–10 wk old) mice were mixed with 5 × 10^5^ CD45.1^+^ WT BM cells and transplanted into lethally irradiated CD45.1^+^/CD45.2^+^ recipients. 8 wk after transplantation, the recipients received six doses of pIpC. (N) Percentage of donor-derived CD45.2^+^ cells in PB. *n* = 5–10 recipients per genotype. *n* = 2 donors per genotype. (O) Percentage of CD45.2^+^ cells in the Lin^+^, Lin^−^, LK, and LSK cell compartments of the recipient mice 11 wk after pIpC treatment. *n* = 7–8 recipients per genotype. Data are mean ± SEM. *, P < 0.05; **, P < 0.01; ***, P < 0.001; ****, P < 0.0001 (Mann-Whitney *U* test).

*Fh1* is uniformly expressed in mouse Lin^–^Sca-1^+^c-Kit^+^ (LSK) CD48^−^CD150^+^ HSCs, LSKCD48^−^CD150^−^ multipotent progenitors, primitive hematopoietic progenitor cells (HPCs; i.e., LSKCD48^+^CD150^−^ HPC-1 and LSKCD48^+^CD150^+^ HPC-2 populations), and Lin^−^Sca-1^−^c-Kit^+^ (LK) myeloid progenitors sorted both from the FL (the major site of definitive hematopoiesis during development) of 14.5–days postcoitum (dpc) embryos and adult BM (Fig. 1 A). To determine the requirement for *Fh1* in HSC maintenance and multilineage hematopoiesis, we conditionally deleted *Fh1* specifically within the hematopoietic system shortly after the emergence of definitive HSCs using the *Vav-iCre* deleter strain (de Boer et al., 2003). We bred *Fh1^fl/fl^* mice (Pollard et al., 2007) with *Vav-iCre* mice and found no viable *Fh1^fl/fl^;Vav-iCre* offspring (Table S2). *Fh1^fl/fl^;Vav-iCre* embryos were recovered at 14.5 dpc at normal Mendelian ratios, suggesting fetal or perinatal lethality. FLs isolated from *Fh1^fl/fl^;Vav-iCre* embryos appeared abnormally small and pale indicating severe impairment in FL hematopoiesis (Fig. 1 B). *Fh1* loss from the hematopoietic system was confirmed by the absence of *Fh1* transcripts (Fig. 1 C) in CD45^+^ and c-Kit^+^ hematopoietic cells from *Fh1^fl/fl^;Vav-iCre* FLs and absence of Fh1 protein in FL c-Kit^+^ cells from *Fh1^fl/fl^;Vav-iCre* embryos (Fig. 1 D). Whereas *Fh1^fl/fl^;Vav-iCre* FLs had decreased numbers of hematopoietic cells because of reduced numbers of differentiated lineage^+^ (Lin^+^) cells, the numbers of primitive FL Lin^−^ cells remained unchanged (Fig. 1 E). Colony-forming cell (CFC) assays indicated the failure of *Fh1*-deficient FL cells to differentiate (Fig. 1 F). Analyses of erythroid differentiation revealed a block of erythropoiesis resulting in severe anemia (Fig. 1 G). *Fh1* is therefore essential for multilineage differentiation of FL stem and/or progenitor cells.

### *Fh1* is essential for HSC maintenance

Next, we asked whether *Fh1* is required for the maintenance of the stem and progenitor cell compartments in FLs. FLs from *Fh1^fl/fl^;Vav-iCre* embryos had normal absolute numbers of LK myeloid progenitors (Fig. 1 H) and LSK stem and primitive progenitor cells (Fig. 1 I) but displayed an increase in total numbers of HSCs compared with control FLs (Fig. 1 J). To test the repopulation capacity of *Fh1*-deficient HSCs, we transplanted 100 CD45.2^+^ HSCs sorted from 14.5-dpc FLs into lethally irradiated syngeneic CD45.1^+^/CD45.2^+^ recipients and found that *Fh1*-deficient HSCs failed to reconstitute short-term and long-term hematopoiesis (Fig. 1, K and L). To test the possibility that *Fh1*-deficient FLs contain stem cell activity outside the immunophenotypically defined LSKCD48^−^CD150^+^ HSC compartment, we transplanted unfractionated FL cells from *Fh1^fl/fl^;Vav-iCre* and control embryos into lethally irradiated recipient mice (together with support BM cells) and found that *Fh1*-deficient FL cells failed to repopulate the recipients (Fig. 1 M). Thus, *Fh1* is dispensable for HSC survival and expansion in the FL but is critically required for HSC maintenance upon transplantation.

To establish the requirement for *Fh1* in adult HSC maintenance, we generated *Fh1^fl/fl^;Mx1-Cre* mice in which efficient recombination is induced by treatment with polyinosinic:polycytidylic acid (pIpC; Kühn et al., 1995). We mixed CD45.2^+^ BM cells from untreated *Fh1^fl/fl^;Mx1-Cre*, *Fh1^+/fl^;Mx1-Cre*, or control mice with CD45.1^+^ BM cells, transplanted them into recipient mice, and allowed for efficient reconstitution (Fig. 1 N). pIpC administration to the recipients of *Fh1^fl/fl^;Mx1-Cre* BM cells resulted in a progressive decline of donor-derived CD45.2^+^ cell chimerism in PB (Fig. 1 N) and a complete failure of *Fh1*-deficient cells to contribute to primitive and mature hematopoietic compartments of the recipients (Fig. 1 O). Therefore, *Fh1* is critical for the maintenance of both FL and adult HSCs.

### *Fh1* deficiency results in cellular fumarate accumulation and decreased maximal mitochondrial respiration

**Figure 2.**
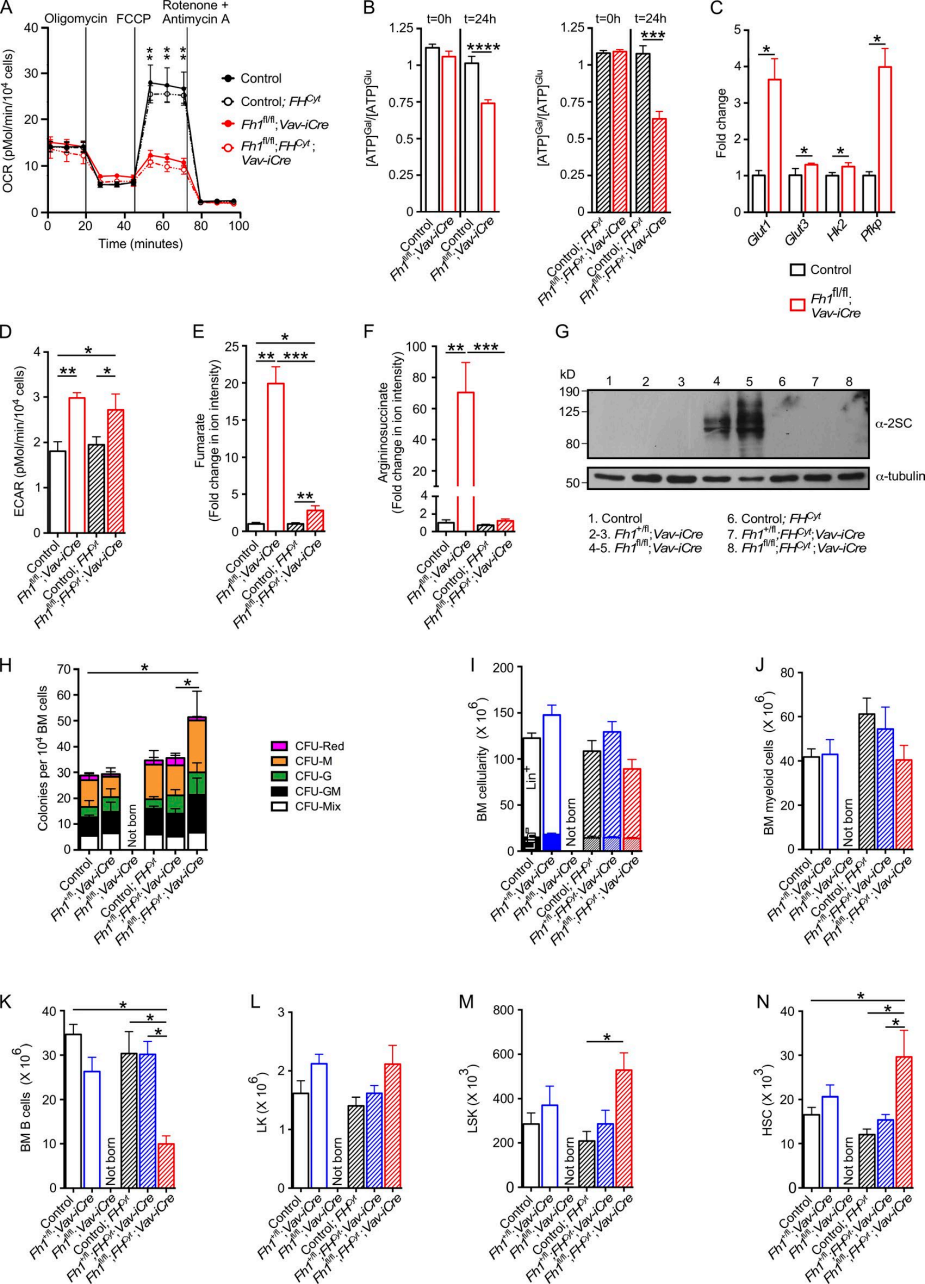
**Cytosolic isoform of Fh1 restores normal steady-state hematopoiesis in *Fh1^fl/fl^;Vav-iCre* mice.** (A) OCR in FL c-Kit^+^ cells under basal conditions and after the sequential addition of oligomycin, FCCP, and rotenone and antimycin A. Control, *n* = 5; *Fh1^fl/fl^;Vav-iCre*, *n* = 3; control;*FH^Cyt^*, *n* = 10; *Fh1^fl/fl^;FH^Cyt^;Vav-iCre*, *n* = 5. (B) Oxidative phosphorylation–dependent ATP production in galactose (Gal)-treated FL c-Kit^+^ *Fh1^fl/fl^;Vav-iCre* and *Fh1^fl/fl^;FH^Cyt^;Vav-iCre* cells. FL c-Kit^+^ cells were cultured in DMEM supplemented with either 25 mM glucose (Glu) or 25 mM Gal. The graph shows the ratio of ATP produced in the presence of Gal (permissive for oxidative phosphorylation only) to ATP generated in the presence of Glu (permissive for both oxidative phosphorylation and glycolysis). Control, *n* = 9; *Fh1^fl/fl^;Vav-iCre*, *n* = 9; control;*FH^Cyt^*, *n* = 6; *Fh1^fl/fl^*;*FH^Cyt^*;Vav-iCre, *n* = 10. (C) Relative expression (normalized to *Actb*) of genes involved in glycolysis in FL c-Kit^+^ cells. *n* = 4–5 per genotype. (D) ECAR under basal conditions in 14.5-dpc FL c-Kit^+^ cells. Control, *n* = 6; *Fh1^fl/fl^;Vav-iCre*, *n* = 5; control;*FH^Cyt^*, *n* = 10; *Fh1^fl/fl^*;*FH^Cyt^*;Vav-iCre, *n* = 4. (E and F) Fumarate (E) and argininosuccinate (F) levels in FL c-Kit^+^ cells measured using LC-MS. Control, *n* = 6; *Fh1^fl/fl^;Vav-iCre*, *n* = 4; control;*FH^Cyt^*, *n* = 6; *Fh1^fl/fl^;FH^Cyt^;Vav-iCre*, *n* = 13. (G) 14.5-dpc FL cell extracts were immunoblotted with a polyclonal anti-2SC antibody. α-Tubulin was used as a loading control. Data are representative of two independent experiments. (H) CFU assays performed with BM cells from 8–10-wk-old mice of the indicated genotypes. CFU-red, CFU-erythroid and/or megakaryocyte; CFU-G, CFU-granulocyte; CFU-M, CFU-monocyte/macrophage; CFU-GM, CFU–granulocyte and monocyte/macrophage; CFU-Mix, at least three of the following: granulocyte, erythroid, monocyte/macrophage, and megakaryocyte. *n* = 3–5 per genotype and are representative of three independent experiments. (I) Total number of BM nucleated cells obtained from two tibias and two femurs of 8–10-wk-old mice. *n* = 3–4 per genotype. (J–N) Total numbers of CD11b^+^Gr-1^+^ myeloid cells (J), CD19^+^B220^+^ B cells (K), LK cells (L), LSK cells (M), and HSCs (N) in two tibias and two femurs. *n* = 3–4 per genotype. Data are mean ± SEM. *, P < 0.05; **, P < 0.01; ***, P < 0.001; ****, P < 0.0001 (Mann-Whitney *U* test).

We next investigated the biochemical consequences of *Fh1* deletion in primitive hematopoietic cells. To investigate how *Fh1* loss affects the oxidative phosphorylation capacity of primitive FL c-Kit^+^ hematopoietic cells, we measured oxygen consumption rate (OCR) under basal conditions and in response to sequential treatment with oligomycin (ATPase inhibitor), carbonyl cyanide-4-(trifluoromethoxy)phenylhydrazone (FCCP; mitochondrial uncoupler), and a concomitant treatment with rotenone and antimycin A (complex I and III inhibitors, respectively). The basal OCR was not affected in *Fh1*-deficient FL c-Kit^+^ cells, suggesting that the majority of mitochondrial NADH (nicotinamide adenine dinucleotide reduced) required for oxygen consumption in these cells originates from TCA-independent sources. However, the maximal OCR (after treatment with FCCP), which reflects maximal mitochondrial respiration, was profoundly decreased in *Fh1*-deficient cells (Fig. 2 A; but not in *Fh1^+/fl^;Vav-iCre* cells; not depicted), indicating that *Fh1* deficiency may result in a compromised capacity to meet increased energy demands associated with metabolic stress or long-term survival (Yadava and Nicholls, 2007; Ferrick et al., 2008; Choi et al., 2009; van der Windt et al., 2012; Keuper et al., 2014). We also found that primitive *Fh1*-deficient FL hematopoietic cells failed to maintain ATP synthesis upon galactose-mediated inhibition of glycolysis (Fig. 2 B, left), consistent with an increased reliance on glycolysis for ATP production. Furthermore, *Fh1*-deficient c-Kit^+^ cells had increased expression of glucose transporters (*Glut1* and *Glut3*) and key glycolytic enzymes *Hk2* and *Pfkp* and displayed increased extracellular acidification rate (ECAR), indicative of enhanced glycolysis (Fig. 2, C–D). Thus, *Fh1* deficiency results in an increase in glycolytic flux and impaired maximal mitochondrial respiration.

To determine the impact of *Fh1* deletion on fumarate levels, we performed mass spectrometry analyses. *Fh1*-deficient FL c-Kit^+^ cells accumulated high levels of endogenous cellular fumarate (Fig. 2 E), consistent with previous observations in nonhematopoietic tissues harboring *Fh1* mutation (Adam et al., 2013). Under the conditions of elevated fumarate, argininosuccinate is generated from arginine and fumarate by the reversed activity of the urea cycle enzyme argininosuccinate lyase (Zheng et al., 2013). We found that argininosuccinate is produced at high levels in *Fh1*-deficient c-Kit^+^ cells (Fig. 2 F). Furthermore, when accumulated at high levels, fumarate modifies cysteine residues in many proteins, forming S-(2-succinyl)-cysteine (2SC; Alderson et al., 2006; Adam et al., 2011; Bardella et al., 2011; Ternette et al., 2013). *Fh1*-deficient c-Kit^+^ cells exhibited high immunoreactivity for 2SC (Fig. 2 G). Thus, primitive hematopoietic cells lacking *Fh1* have compromised maximal mitochondrial respiration, display increased glycolysis, fail to maintain normal ATP production upon inhibition of glycolysis, and accumulate high levels of fumarate resulting in excessive protein succination.

### Efficient fumarate metabolism is essential for HSC maintenance and multilineage hematopoiesis

Mechanistically, the phenotypes observed upon *Fh1* deletion could result from the genetic block in the TCA cycle or the accumulation of cellular fumarate (Pollard et al., 2007; Adam et al., 2011). To differentiate between these two mechanisms, we used mice ubiquitously expressing a human cytoplasmic isoform of FH (FH^Cyt^, which lacks the mitochondrial targeting sequence and therefore is excluded from the mitochondria; Adam et al., 2013). FH^Cyt^ does not restore defects in mitochondrial oxidative metabolism but normalizes levels of total cellular fumarate (O’Flaherty et al., 2010; Adam et al., 2013). Although primitive hematopoietic cells from FLs of *Fh1^fl/fl^;FH^Cyt^;Vav-iCre* embryos had normal mitochondrial membrane potential (not depicted), they displayed defective maximal respiration (Fig. 2 A) and impaired compensatory mitochondrial ATP production upon inhibition of glycolysis (Fig. 2 B, right), as well as an increase in ECAR (Fig. 2 D) similar to *Fh1^fl/fl^;Vav-iCre* FL cells. Furthermore, primitive *Fh1^fl/fl^;FH^Cyt^;Vav-iCre* FL cells had significantly reduced levels of cellular fumarate (Fig. 2 E) and argininosuccinate (Fig. 2 F) and undetectable immunoreactivity to 2SC (Fig. 2 G), indicating that the biochemical consequences of fumarate accumulation were largely abolished by the *FH^Cyt^* transgene expression. Although *FH^Cyt^* transgene decreased overall cellular levels of fumarate, argininosuccinate, and succinated proteins, we cannot exclude the possibility that fumarate is elevated in mitochondria and contributes to the impairment of mitochondrial function in the absence of mitochondrial Fh1. Collectively, although cells from *Fh1^fl/fl^;FH^Cyt^;Vav-iCre* FLs displayed impaired maximal respiration, they had cellular fumarate levels comparable with control cells.

Notably, *Fh1^fl/fl^;FH^Cyt^;Vav-iCre* mice were born at normal Mendelian ratios (Table S3) and matured to adulthood without any obvious defects. BM cells from adult *Fh1^fl/fl^;FH^Cyt^;Vav-iCre* mice efficiently generated myeloid colonies (Fig. 2 H), had normal BM cellularity (Fig. 2 I), and displayed multilineage hematopoiesis (Fig. 2, J and K), despite reduced numbers of B cells (Fig. 2 K). Furthermore, they had unaffected numbers of LK myeloid progenitor cells (Fig. 2 L) and increased numbers of LSK cells (Fig. 2 M) and HSCs (Fig. 2 N). Therefore, it is critical that HSCs and/or primitive progenitor cells efficiently metabolize fumarate to sustain hematopoietic differentiation. Finally, HSCs that acquire mitochondrial Fh1 deficiency (which abolishes maximal mitochondrial respiration) shortly after their emergence manage to survive, expand in the FL, colonize the BM, and sustain steady-state multilineage hematopoiesis, implying that mitochondrial Fh1 is largely dispensable for these processes.

### Mitochondrial Fh1 deficiency compromises HSC self-renewal

**Figure 3.**
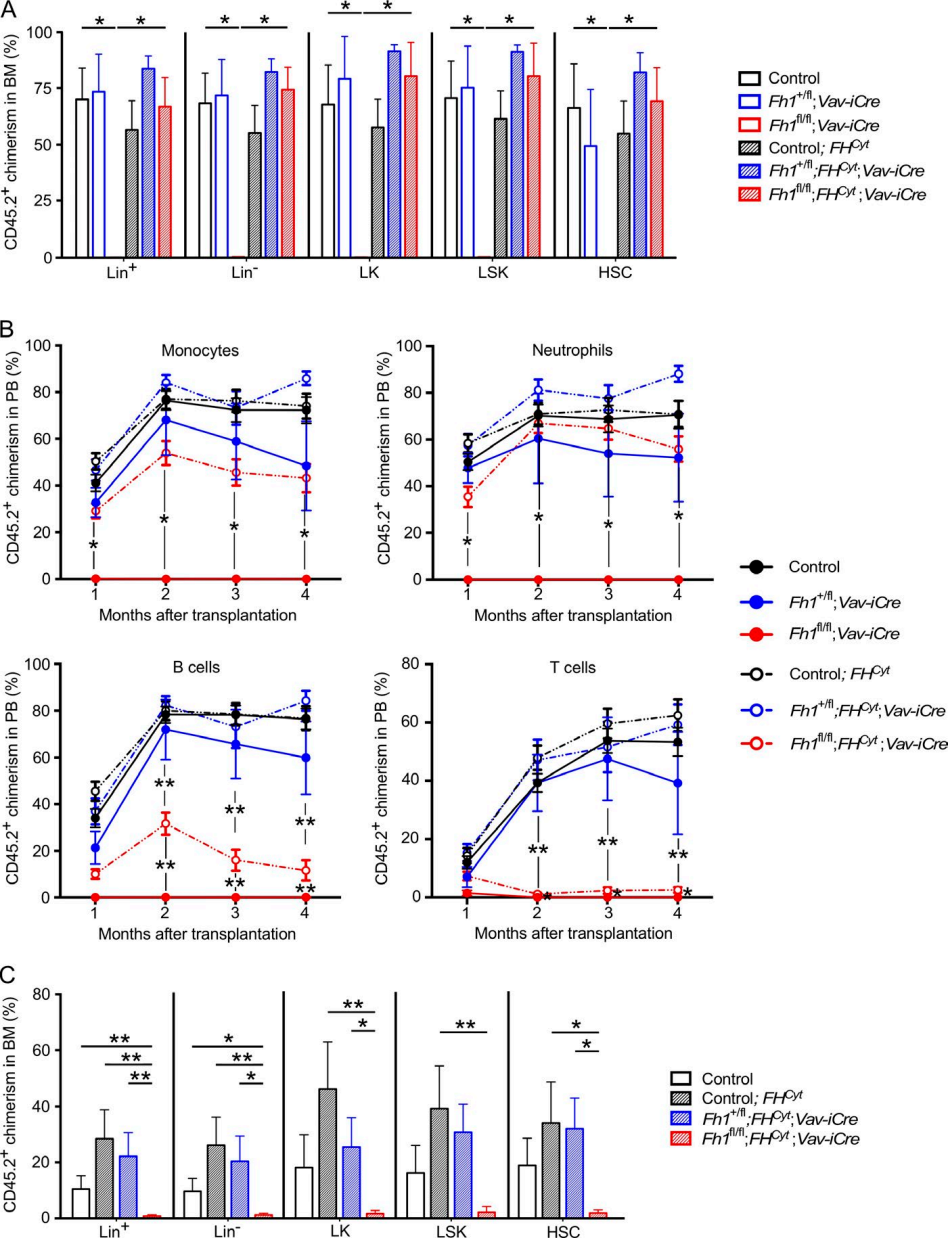
**Mitochondrial Fh1 is essential for HSC self-renewal.** (A–C) 100 FL HSCs were transplanted into lethally irradiated 8–10-wk-old C57BL/6 CD45.1^+^/CD45.2^+^ recipient mice together with 2 × 10^5^ CD45.1^+^ syngeneic competitor BM cells. The primary recipients were analyzed 20 wk after transplantation. 2,000 CD45.2^+^LSK cells were sorted from their BM and transplanted into secondary recipients together with competitor BM cells. Secondary recipients were analyzed 20 wk after transplantation. (A) Percentage of CD45.2^+^ cells in the Lin^+^, Lin^−^, LK, LSK, and HSC compartments in the BM of primary recipients. *n* = 4–5 recipients per donor. (B) Percentage of CD45.2^+^ cells in the monocyte, neutrophil, B cell, and T cell compartments in the PB of primary recipients. *n* = 4–5 recipients per donor. Number of donors used in A and B: control, *n* = 6; *Fh1^+/fl^;Vav-iCre*, *n* = 2; *Fh1^fl/fl^;Vav-iCre*, *n* = 2; control;*FH^Cyt^*, *n* = 6; *Fh1^+/fl^;FH^Cyt^;Vav-iCre*, *n* = 3; *Fh1^fl/fl^;FH^Cyt^;Vav-iCre*, *n* = 3. (C) Percentage of CD45.2^+^ cells in the Lin^+^, Lin^−^, LK, LSK, and HSC compartments in the BM of the secondary recipients. *n* = 4–5 recipients per donor. Number of donors: control, *n* = 3; control;*FH^Cyt^*, *n* = 4; *Fh1^+/fl^;FH^Cyt^;Vav-iCre*, *n* = 2; *Fh1^fl/fl^;FH^Cyt^;Vav-iCre*, *n* = 3. Data are mean ± SEM. *, P < 0.05; **, P < 0.01 (Mann-Whitney *U* test).

To stringently test the long-term self-renewal capacity of HSCs lacking mitochondrial *Fh1*, we performed serial transplantation assays. We transplanted 100 HSCs from FLs of *Fh1^fl/fl^;FH^Cyt^;Vav-iCre*, *Fh1^fl/fl^;Vav-iCre*, and control 14.5-dpc embryos together with 200,000 support BM cells. Whereas *Fh1^fl/fl^;Vav-iCre* HSCs failed to repopulate the recipients, *Fh1^fl/fl^;FH^Cyt^;Vav-iCre* HSCs contributed to primitive and more mature hematopoietic compartments of the recipient mice (Fig. 3 A). Primary recipients of *Fh1^fl/fl^;FH^Cyt^;Vav-iCre* HSCs displayed efficient myeloid lineage reconstitution, whereas B and T lymphoid–lineage reconstitution was less robust (Fig. 3 B), suggesting that mitochondrial Fh1 is required for lymphoid cell differentiation or survival. Next, we sorted BM LSK cells from the primary recipients and retransplanted them into secondary recipients. *Fh1^fl/fl^;FH^Cyt^;Vav-iCre* HSCs failed to contribute to the BM hematopoietic compartments of the recipients 20 wk after transplantation (Fig. 3 C). Therefore, HSCs lacking mitochondrial Fh1 display progressive loss of self-renewal potential upon serial transplantation.

### Hematopoietic defects upon *Fh1* deletion are not caused by oxidative stress or the activation of Nrf2-dependent pathways

**Figure 4.**
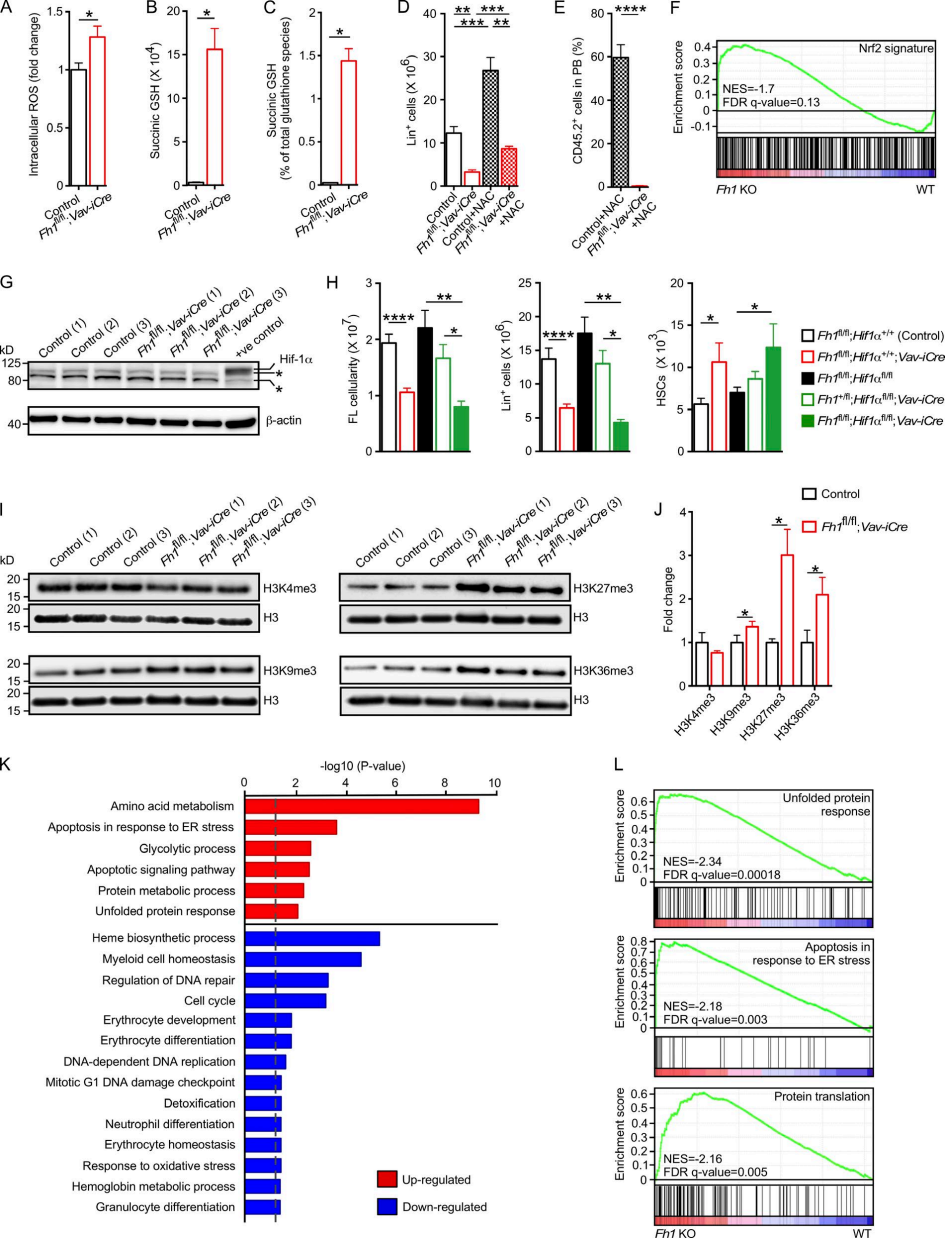
**Molecular consequences of *Fh1* deletion in primitive hematopoietic cells.** (A) Intracellular ROS in FL c-Kit^+^ cells. The mean of mean fluorescence intensities ± SEM is shown. Control, *n* = 6; *Fh1^fl/fl^;Vav-iCre*, *n* = 3. (B and C) GSH species in 14.5-dpc FL c-Kit^+^ cells measured using LC-MS. Succinic GSH levels (arbitrary units; B) and percentage of Succinic GSH within the total GSH species (C) are shown. Control, *n* = 6; *Fh1^fl/fl^;Vav-iCre*, *n* = 3. (D and E) Pregnant females were treated with NAC administered 7 d before the embryo harvest. (D) Lin^+^ cell numbers in 14.5-dpc FLs. Control, *n* = 15; *Fh1^fl/fl^;Vav-iCre*, *n* = 6; control + NAC, *n* = 4; *Fh1^fl/fl^;Vav-iCre* + NAC, *n* = 4. (E) 600,000 total FL cells of 14.5-dpc embryos from NAC-treated pregnant females were transplanted into lethally irradiated CD45.1^+^/CD45.2^+^ recipient mice together with 200,000 CD45.1^+^ competitor BM cells. Recipients were continuously treated with NAC. Data represent percentage of donor-derived CD45.2^+^ cells in PB 3 wk after transplantation. *n* = 10–11 recipients per genotype. *n* = 4 donors per genotype. (F) GSEA showing that the Nrf2 signature is not significantly affected in *Fh1*-deficient (*Fh1* KO) FL Lin^−^c-Kit^+^ cells. FDR, false discovery rate; NES, normalized enrichment score. (G) Western blots for Hif-1α and β-actin in c-Kit^+^ cells from 14.5-dpc FLs. *n* = 3 per genotype. CoCl_2_-treated FL c-Kit^+^ cells were used as a positive control for Hif-1α. Asterisks indicate nonspecific bands. (H) Total FL cellularity and total number of Lin^+^ cells and HSCs in 14.5-dpc FLs. *Fh1^fl/fl^;Hif-1α^+/+^*, *n* = 12; *Fh1^fl/fl^;Hif-1α^+/+^;Vav-iCre*, *n* = 9; *Fh1^fl/fl^;Hif-1α^fl/fl^*, *n* = 6; *Fh1^+/fl^;Hif-1α^fl/fl^;Vav-iCre*, *n* = 5; *Fh1^fl/fl^;Hif-1α^fl/fl^;Vav-iCre*, *n* = 4. (I) Western blot for H3K4me3, H3K9me3, H3K27me3, H3K36me3, and total H3 in 14.5-dpc FL c-Kit^+^ cells. *n* = 3 per genotype. (J) Quantification of the data (normalized to total H3) shown in panel I. *n* = 3 per genotype. (K) Biological processes (presented as –log10 [p-value]) that are enriched in up-regulated and down-regulated genes in *Fh1*-deficient FL Lin^−^c-Kit^+^ cells versus control cells. Analysis was performed using the Gene Ontology Consortium database. The dashed gray line indicates P = 0.05. (L) Signature enrichment plots from GSEA analyses using unfolded protein response, apoptosis in response to ER stress, and protein translation signature gene sets. (F, K, and L) Gene expression analysis was performed using Lin^−^c-Kit^+^ cells from three *Fh1^fl/fl^* (WT) and four *Fh1^fl/fl^;Vav-iCre* (*Fh1* KO) embryos. Data are mean ± SEM. *, P < 0.05; **, P < 0.01; ***, P < 0.001; ****, P < 0.0001 (Mann-Whitney *U* test).

Because elevated cellular fumarate is a major cause of hematopoietic defects in *Fh1^fl/fl^;Vav-iCre* FLs, we next explored potential mechanisms through which fumarate impairs FL hematopoiesis. In nonhematopoietic tissues, fumarate succinates cysteine residues of biologically active molecules (including glutathione [GSH]; Sullivan et al., 2013; Zheng et al., 2015) and numerous proteins (Adam et al., 2011; Ternette et al., 2013). Elevated fumarate causes oxidative stress by succinating GSH and, thus, generating succinic GSH and depleting the GSH pool (Sullivan et al., 2013; Zheng et al., 2015). We found that reactive oxygen species (ROS) were modestly increased in *Fh1^fl/fl^;Vav-iCre* FL c-Kit^+^ cells compared with control cells (Fig. 4 A). The quantity of succinic GSH was elevated in *Fh1^fl/fl^;Vav-iCre* FL c-Kit^+^ cells (Fig. 4 B), but succinic GSH constituted only ∼1.5% of the total pool of GSH species (Fig. 4 C). Furthermore, we found that administration of the antioxidant *N*-acetylcysteine (NAC) to timed-mated pregnant females did not rescue the reduced FL cellularity (not depicted) and failed to reverse decreased numbers of Lin^+^ FL cells (Fig. 4 D). Finally, HSCs sorted from NAC-treated *Fh1^fl/fl^;Vav-iCre* FLs failed to reconstitute hematopoiesis in NAC-treated recipient mice (Fig. 4 E). Therefore, oxidative stress caused by GSH depletion does not cause hematopoietic defects resulting from fumarate accumulation.

Loss of *Fh1* in renal cysts is associated with up-regulation of the Nrf2-mediated antioxidant response pathway because of fumarate-mediated succination of Keap1, which normally promotes Nrf2 degradation (Adam et al., 2011). However, the analyses of global gene expression profiling of FL Lin^−^c-Kit^+^ primitive hematopoietic cells from 14.5-dpc *Fh1^fl/fl^;Vav-iCre* and control embryos revealed no significant enrichment for Nrf2 signature (Fig. 4 F). Thus, the activation of the Nrf2-dependent pathways is not responsible for defective hematopoiesis in *Fh1^fl/fl^;Vav-iCre* FLs.

### *Fh1* deficiency in primitive hematopoietic cells has no impact on the Hif-1–dependent pathways and does not affect global 5-hydroxymethylcytosine levels

Fumarate is known to competitively inhibit 2-oxoglutarate (2OG)–dependent oxygenases including Hif prolyl hydroxylase Phd2 resulting in stabilization of Hif-1α (Adam et al., 2011). Given that *Phd2* deletion and stabilization of Hif-1α results in HSC defects (Takubo et al., 2010; Singh et al., 2013), we asked whether elevated fumarate increases the Hif-1α protein levels upon *Fh1* deletion in primitive hematopoietic cells. We found that Hif-1α protein was undetectable in *Fh1^fl/fl^;Vav-iCre* and control FL c-Kit^+^ cells (Fig. 4 G), and additional deletion of *Hif-1α* in *Fh1*-deficient embryos failed to rescue embryonic lethality (not depicted), restore total FL cellularity, reverse decreased FL Lin^+^ cell numbers, or normalize elevated numbers of FL HSCs (Fig. 4 H). Thus, Hif-1α is not involved in generating hematopoietic defects in *Fh1^fl/fl^;Vav-iCre* FLs. Given that fumarate can inhibit the Tet family of 5-methylcytosine hydroxylases (Xiao et al., 2012), we measured the levels of 5-hydroxymethylcytosine in *Fh1^fl/fl^;Vav-iCre* and control c-Kit^+^ cells and did not find any differences (not depicted), suggesting that fumarate-mediated Tet inhibition does not play a major role in generating hematopoietic defects in *Fh1^fl/fl^;Vav-iCre* FLs.

### *Fh1* deletion results in increased histone H3 trimethylation in primitive hematopoietic cells

Emerging evidence indicates that 2OG-dependent JmjC domain–containing histone demethylases (KDMs) play important roles in HSC biology and hematopoiesis (Stewart et al., 2015; Andricovich et al., 2016). Given that fumarate inhibits enzymatic activity of KDMs (Xiao et al., 2012), we examined the abundance of H3K4me3, H3K9me3, H3K27me3, and H3K36me3 in nuclear extracts from FL c-Kit^+^ cells isolated from 14.5-dpc *Fh1^fl/fl^;Vav-iCre* and control embryos. Western blot analyses revealed an increase in levels of H3K9me3, H3K27me3, and H3K36me3 but not H3K4me3 in *Fh1*-deficient cells (Fig. 4, I and J). Although these data suggest that *Fh1* deficiency results in enhanced trimethylation of H3, the identity of KDMs that are inhibited by fumarate and the causal roles for increased H3 trimethylation in mediating HSC and hematopoietic defects upon *Fh1* deletion remain to be elucidated.

### *Fh1* deletion promotes a gene expression signature that facilitates hematopoietic defects

To understand the molecular signatures associated with *Fh1* deficiency, we performed gene expression profiling of FL Lin^−^c-Kit^+^ primitive hematopoietic cells from *Fh1^fl/fl^;Vav-iCre* and control embryos. We found that genes up-regulated in *Fh1*-deficient cells are highly enriched in categories related to apoptosis in response to ER stress, protein metabolic process/protein translation, and unfolded protein response (Fig. 4, K and L). Down-regulated genes are enriched in pathways related to heme biosynthesis, erythroid and myeloid function, and the cell cycle (Fig. 4 K). Although further detailed work will be needed to experimentally verify this, we propose that enhanced ER stress, unfolded protein response, and increased protein translation (which are known to contribute to HSC depletion and hematopoietic failure; Miharada et al., 2014; Signer et al., 2014; van Galen et al., 2014) may be responsible for hematopoietic defects upon *Fh1* deletion.

### *Fh1* deficiency abolishes leukemic transformation and LIC functions

**Figure 5.**
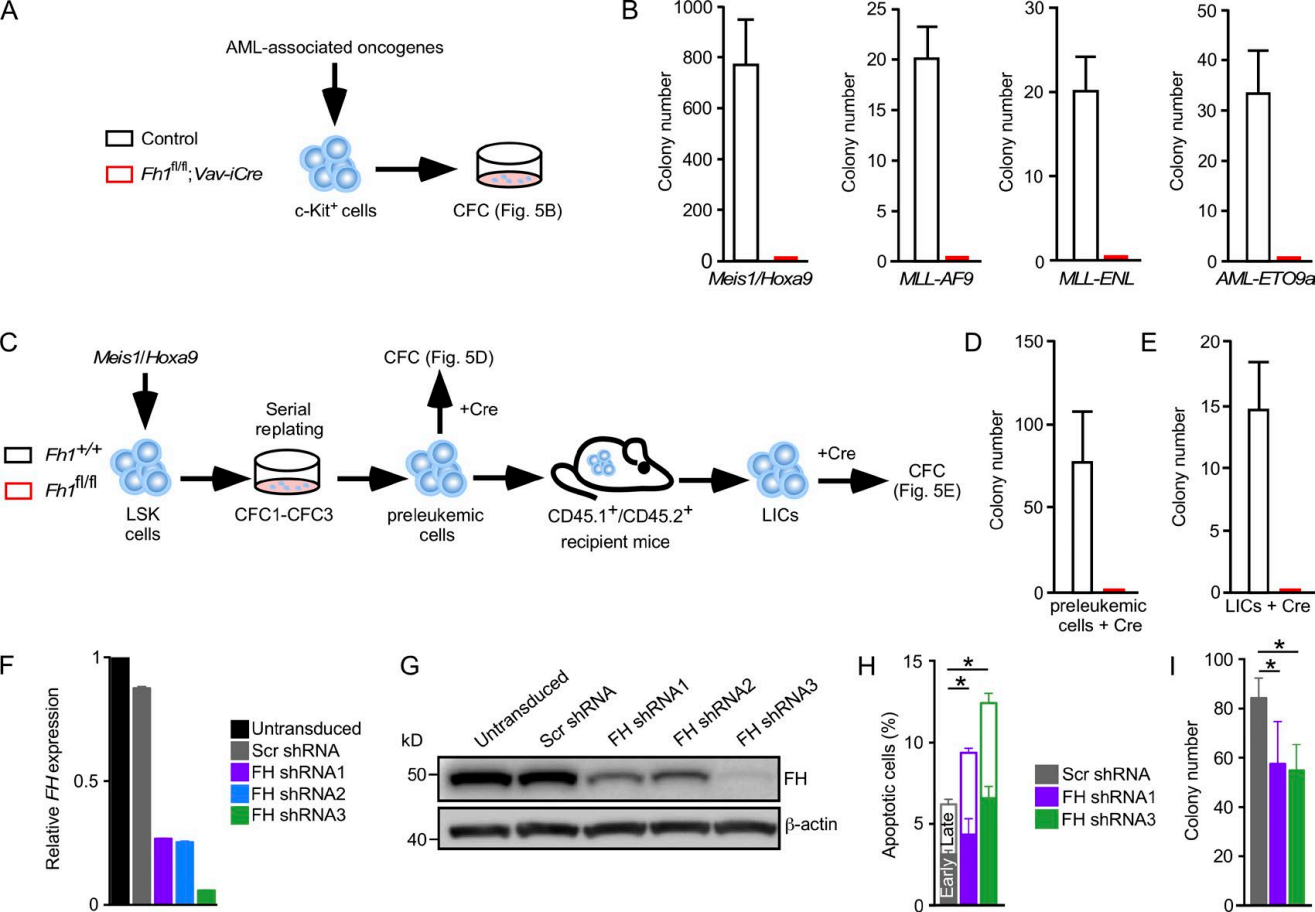
***Fh1* is required for leukemic transformation.** (A) 14.5-dpc FL c-Kit^+^ cells were transduced with *Meis1*/*Hoxa9*, *MLL-AF9*, *MLL-ENL*, and *AML1-ETO9a* retroviruses and plated into methylcellulose. (B) Colony counts 6 d after plating are shown. *n* = 4–5 per genotype. (C) *Fh1^+/+^* and *Fh1^fl/fl^* (without *Vav-iCre*) FL LSK cells were co-transduced with *Meis1* and *Hoxa9* retroviruses and serially replated. The cells were subsequently infected with a bicistronic lentivirus expressing *iCre* and a Venus reporter. Venus^+^ cells were plated into methylcellulose. In parallel, *Fh1^+/+^* and *Fh1^fl/fl^* preleukemic cells were transplanted into recipient mice. LICs (CD45.2^+^c-Kit^+^ cells) were sorted from the BM of leukemic recipients, transduced with *Cre* lentivirus, and plated into methylcellulose. (D) Number of colonies generated by *Cre*-expressing preleukemic cells. *n* = 3 per genotype. (E) Number of colonies generated by *Cre*-expressing LICs. *n* = 3 per genotype. (F) Relative levels of *FH* mRNA (normalized to *ACTB*) in untransduced THP-1 cells and THP-1 cells transduced with lentiviruses expressing scrambled shRNA (Scr shRNA) and three different shRNAs targeting *FH* (FH shRNA1, FH shRNA2, and FH shRNA3). *n* = 3. (G) Western blot for FH and β-actin in THP-1 cells described in Fig. 5 F. (H) Apoptosis assays performed with THP-1 cells transduced with lentiviruses expressing scrambled shRNA, FH shRNA1, and FH shRNA3. The graph depicts the percentage of annexin V^+^DAPI^−^ cells in early apoptosis and annexin V^+^DAPI^+^ in late apoptosis. *n* = 4. (I) CFC assays with THP-1 cells expressing scrambled shRNA, FH shRNA1, and FH shRNA3. *n* = 5. Data are mean ± SEM. *, P < 0.05 (Mann-Whitney *U* test).

Although *FH* is a tumor suppressor (i.e., *FH* mutations result in hereditary leiomyomatosis and renal-cell cancer) and fumarate is proposed to function as an oncometabolite (Yang et al., 2013), the role of FH in leukemic transformation remains unknown. Given that human acute myeloid leukemia (AML) cells express high levels of FH protein (López-Pedrera et al., 2006; Elo et al., 2014) and enzymatic activity of FH is increased in AML samples compared with cells from normal controls (Tanaka and Valentine, 1961), we next investigated the role for *Fh1* in leukemic transformation. We used a mouse model of AML in which the development and maintenance of LICs is driven by *Meis1* and *Hoxa9* oncogenes (Wang et al., 2010; Vukovic et al., 2015). *Meis1* and *Hoxa9* are frequently overexpressed in several human AML subtypes (Lawrence et al., 1999; Drabkin et al., 2002), and their overexpression in mouse hematopoietic stem and progenitor cells generates self-renewing LICs (Kroon et al., 1998). In the *Meis1*/*Hoxa9* model used here, the FL LSK or c-Kit^+^ cell populations are transduced with retroviruses expressing *Meis1* and *Hoxa9* and are serially replated, generating a preleukemic cell population, which, upon transplantation to primary recipients, develops into LICs causing AML. LICs are defined by their capacity to propagate AML with short latency in secondary recipients (Somervaille and Cleary, 2006; Yeung et al., 2010; Vukovic et al., 2015). We found that *Fh1^fl/fl^;Vav-iCre* FL stem and progenitor cells transduced with *Meis1*/*Hoxa9* (Fig. 5 A) failed to generate colonies in methylcellulose (Fig. 5 B). To corroborate these findings, we used retroviruses expressing *MLL* fusions which are frequently found in acute monoblastic leukemia (AML M5) and are associated with an unfavorable prognosis in AML, namely *MLL-ENL* (fusion oncogene resulting from t[11;19]) and *MLL-AF9* (resulting from t[9;11]; Krivtsov and Armstrong, 2007; Lavallée et al., 2015). We also used *AML1-ETO9a*, a splice variant of AML1-ETO that is frequently expressed in t(8;21) patients with AML M2, and its high expression correlates with poor AML prognosis (Jiao et al., 2009). MLL fusions and *AML1-ETO9a* drive leukemogenesis through distinct pathways and are frequently used to transform mouse hematopoietic cells (Zuber et al., 2009; Smith et al., 2011; Velasco-Hernandez et al., 2014). We found that *Fh1*-deficient cells transduced with *MLL-ENL*, *MLL-AF9*, and *AML1-ETO9a* were unable to generate colonies (Fig. 5 B), indicating the requirement for *Fh1* in in vitro transformation. Next, we determined the impact of *Fh1* deletion on the colony formation capacity of preleukemic cells (Fig. 5 C). We transduced *Fh1^+/+^* and *Fh1^fl/fl^* FL LSK cells with *Meis1* and *Hoxa9* retroviruses, and after three rounds of replating, we infected the transformed cells with *Cre* lentiviruses. *Fh1^fl/fl^* cells expressing *Cre* failed to generate colonies in CFC assays (Fig. 5 D). Thus, *Fh1* deletion inhibits the generation of preleukemic cells and abolishes their clonogenic capacity.

To determine the requirement for *Fh1* in LICs, LSK cells from *Fh1^+/+^* and *Fh1^fl/fl^* FLs were transduced with *Meis1* and *Hoxa9* retroviruses and transplanted into primary recipients (Fig. 5 C). LICs isolated from leukemic primary recipient mice were infected with *Cre* lentivirus and plated into methylcellulose. We found that *Fh1*-deficient LICs were unable to generate colonies (Fig. 5 E). Next, we investigated the requirement for *FH* in human established leukemic cells by knocking down the expression of *FH* in human AML (M5) THP-1 cells harboring MLL-AF9 translocation. We generated lentiviruses expressing three independent short hairpins (i.e., *FH* shRNA 1–3) targeting *FH* and a scrambled shRNA sequence. Based on knockdown efficiency (Fig. 5, F and G), we selected *FH* shRNA1 and *FH* shRNA3 for further experiments. *FH* knockdown increased apoptosis of THP-1 cells (Fig. 5 H) and decreased their ability to form colonies (Fig. 5 I). Therefore, *Fh1* is required for survival of both mouse LICs and human established leukemic cells. Finally, we conclude that the tumor suppressor functions of *FH* are tissue specific and do not extend to hematopoietic cells.

### Mitochondrial Fh1 is necessary for AML development but is not required for disease maintenance

**Figure 6.**
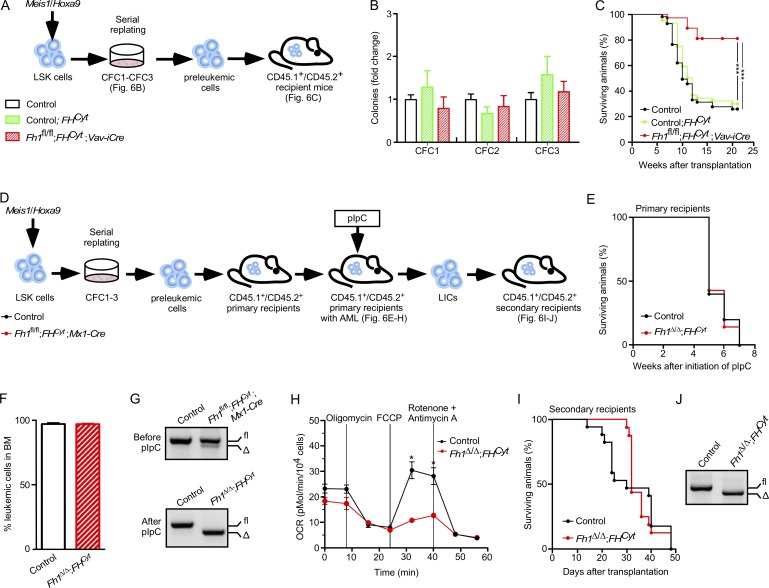
**Mitochondrial Fh1 is necessary for efficient leukemia establishment but is not required for AML propagation.** (A) Control, *Fh1^fl/fl^;FH^Cyt^* (i.e., Control;*FH^Cyt^*), and *Fh1^fl/fl^;FH^Cyt^;Vav-iCre* FL LSK cells were co-transduced with *Meis1* and *Hoxa9* retroviruses and serially replated. 100,000 c-Kit^+^ preleukemic cells were transplanted into sublethally irradiated recipient mice. (B) CFC counts at each replating. Data are mean ± SEM. *n* = 6–8 per genotype. (C) Kaplan-Meier survival curve of primary recipient mice. *n* = 8–10 recipients per genotype and 4 donors per genotype. ***, P < 0.001 (log-rank [Mantel-Cox] test). (D) Control and *Fh1^fl/fl^;FH^Cyt^;Mx1-Cre* FL LSK cells were co-transduced with *Meis1* and *Hoxa9* retroviruses and serially replated. The resultant preleukemic cells were transplanted into sublethally irradiated recipients. Once leukemic CD45.2^+^ cells reached 20% in the PB of recipient mice, the recipients received eight doses of pIpC. 10,000 LICs (CD45.2^+^c-Kit^+^) from primary recipients were transplanted into secondary recipients. (E) Kaplan-Meier survival curve of primary recipient mice. pIpC treatment was initiated 5 wk after transplantation. *n* = 5–7 recipients per genotype. (F) Percentage of CD45.2^+^ cells in BM of primary recipient mice with terminal leukemia. Data are mean ± SEM. *n* = 5–7 recipients per genotype. (G) Genomic PCR assessing *Fh1* deletion before pIpC (top) and after pIpC (bottom) treatment. Δ, excised allele; fl, undeleted conditional allele. (H) OCR in LICs isolated from the BM of primary recipients treated with pIpC. OCR was assayed as described in Fig 2 A. Data are mean ± SEM. *n* = 3–5. *, P < 0.05 (Mann-Whitney *U* test). (I) Kaplan-Meier survival curve of secondary recipients transplanted with LICs sorted from leukemic primary recipients. *n* = 10 per genotype. (J) Representative gel showing PCR amplification of genomic DNA from the total BM of secondary recipients with terminal leukemia.

Next, we investigated the impact of mitochondrial Fh1 deficiency on in vitro transformation and development and maintenance of LICs (Fig. 6, A–C). FL *Fh1^fl/fl^;FH^Cyt^;Vav-iCre* cells transduced with *Meis1*/*Hoxa9* retroviruses had normal serial replating capacity (Fig. 6 B), and the established preleukemic cells had normal proliferative capacity and cell-cycle status (not depicted). Thus, elevated fumarate is largely responsible for the inability of *Fh1^fl/fl^;Vav-iCre* stem and progenitor cells to undergo in vitro transformation. To establish the requirement for mitochondrial Fh1 in AML development in vivo, we transplanted control (*Fh1^fl/fl^*), control;*FH^Cyt^*, and *Fh1^fl/fl^;FH^Cyt^;Vav-iCre Meis1*/*Hoxa9*-transduced preleukemic cells into sublethally irradiated recipient mice (Fig. 6 A). We found that the percentage of recipients of *Fh1^fl/fl^;FH^Cyt^;Vav-iCre* cells that developed terminal AML was significantly reduced (and the disease latency was extended) compared with recipients of control and control;*FH^Cyt^* cells (Fig. 6 C). Finally, OCR measurements in LICs sorted from those recipients of *Fh1^fl/fl^;FH^Cyt^;Vav-iCre* cells that succumbed to AML revealed that *Fh1^fl/fl^;FH^Cyt^;Vav-iCre* LICs had defective maximal mitochondrial respiration compared with control and control;*FH^Cyt^* LICs (not depicted). Thus, mitochondrial Fh1 is required for efficient generation of LICs and AML development in a *Meis1*/*Hoxa9*-driven model of leukemogenesis.

Given that mitochondrial Fh1 was important for leukemia initiation, we next asked whether inducible deletion of mitochondrial Fh1 from leukemic cells impacts on leukemia propagation and LIC maintenance (Fig. 6, D–J). We transduced control and *Fh1^fl/fl^;FH^Cyt^;Mx1-Cre* FL LSK cells with *Meis1*/*Hoxa9* retroviruses, and after serial replating, the resultant preleukemic cells were transplanted into primary recipient mice (Fig. 6 D). Upon disease diagnosis (i.e., 20% of CD45.2^+^ leukemic cells in the PB), the mice received eight pIpC doses (Fig. 6 D). Recipients of both control and *Fh1^fl/fl^;FH^Cyt^;Mx1-Cre* cells equally succumbed to terminal AML (Fig. 6, E and F). After confirming efficient *Fh1* deletion (Fig. 6 G) and defective maximal respiration (Fig. 6 H), we isolated LICs from the BM of leukemic primary recipient mice and transplanted them into secondary recipients. We found that LICs lacking mitochondrial Fh1 and control LICs equally efficiently caused leukemia in secondary recipients (Fig. 6, I and J). Thus, mitochondrial Fh1 is necessary for efficient LIC generation but is not required for their ability to efficiently propagate *Meis1*/*Hoxa9*-driven leukemia.

## Discussion

By performing genetic dissection of multifaceted functions of the key metabolic gene *Fh1*, we have uncovered a previously unknown requirement for fumarate metabolism in the hematopoietic system. We conclude that efficient utilization of intracellular fumarate is required to prevent its potentially toxic effects and is central to the integrity of HSCs and hematopoietic differentiation. Furthermore, although fumarate promotes oncogenesis in the kidney (Yang et al., 2013), it has the opposite effect in the hematopoietic system, i.e., it inhibits leukemic transformation. Our data, indicating a detrimental impact of fumarate on hematopoiesis, collectively with fumarate’s functions as an oncometabolite in nonhematopoietic tumors (Yang et al., 2013), or a protective role within the myocardium (Ashrafian et al., 2012), highlight distinct functions of fumarate in different tissues.

Elevated fumarate within the hematopoietic system is likely to perturb multiple biochemical mechanisms. Fumarate is known to inhibit 2OG-dependent oxygenases, including HIF-hydroxylase Phd2 (Hewitson et al., 2007), the Tet enzymes, and KDMs (Xiao et al., 2012), and as a consequence, tumor cells with FH mutations have increased HIF-1α stability and display a hypermethylator phenotype (Isaacs et al., 2005; Pollard et al., 2007; Letouzé et al., 2013; Castro-Vega et al., 2014). We found that hematopoietic defects resulting from elevated fumarate are most likely generated through the Hif-1–independent and Tet-independent mechanisms. However, consistent with the ability of fumarate to inhibit KDMs, we found that levels of H3K9me3, H3K27me3, and H3K36me3 were elevated in primitive hematopoietic cells lacking *Fh1*. Although detailed underlying mechanisms remain to be elucidated, we propose that elevated fumarate may cause the observed phenotypes by inhibiting these KDMs that are essential for normal hematopoiesis and HSC functions, including KDM5B (Stewart et al., 2015) and KDM2B (Andricovich et al., 2016).

Fumarate is also known to cause succination of cysteine residues of numerous proteins (e.g., Keap1, which normally promotes Nrf2 degradation; Adam et al., 2011; Ternette et al., 2013) or GSH (Sullivan et al., 2013; Zheng et al., 2015). However, our data indicated that hematopoietic defects upon *Fh1* deletion are unlikely to be mediated by Nrf2 activation or GSH depletion. Given our findings that *Fh1*-deficient cells have increased signatures of ER stress and unfolded protein response, it will be of high interest to determine whether increased global protein succination in *Fh1*-deficient cells results in protein misfolding in HSCs, leading to the activation of unfolded protein response, which is detrimental to HSC integrity (van Galen et al., 2014).

*Fh1* deletion with the simultaneous reexpression of cytosolic FH allowed us to investigate the genetic requirement for mitochondrial Fh1 in long-term HSC functions. Our serial transplantation assays revealed that mitochondrial Fh1 was essential for HSC self-renewal, indicating a key role for an intact TCA cycle in HSC maintenance. Intriguingly, although mitochondrial Fh1 deficiency did not affect myeloid output under steady-state conditions and upon transplantation, the lack of mitochondrial Fh1 had an impact on the lymphoid output. These data imply differential requirements for the intact TCA cycle in lineage commitment and/or differentiation of primitive hematopoietic cells, meriting further investigations.

Both self-renewing HSCs and LICs are thought to rely heavily on glycolysis while they suppress the TCA cycle (Simsek et al., 2010; Wang et al., 2014). We used the genetic mitochondrial Fh1 deficiency to examine the differential requirement for mitochondrial Fh1 in long-term HSC self-renewal and the development and maintenance of LICs. We conclude that self-renewing HSCs critically require intact mitochondrial Fh1 and the capacity for maximal mitochondrial respiration to maintain their pool. However, although mitochondrial Fh1 was necessary for LIC development, it had no impact on maintenance of LICs. Thus, we reveal a differential requirement for the mitochondrial TCA enzyme Fh1 in normal hematopoiesis and *Meis1*/*Hoxa9*-driven leukemia propagation. The discovery of mechanisms underlying different metabolic requirements in HSCs and LICs represents a key area for future investigations.

## Materials and methods

### Mice

All mice were on a C57BL/6 genetic background. *Fh1^fl/fl^* (Pollard et al., 2007), *Hif-1α^fl/fl^* (Ryan et al., 2000; Vukovic et al., 2016), and *V5-FH^Cyt^* (referred to as *FH^Cyt^*; Adam et al., 2013) were described previously. *Vav-iCre* and *Mx1-Cre* were purchased from The Jackson Laboratory. All transgenic and knockout mice were CD45.2^+^. Congenic recipient mice were CD45.1^+^/CD45.2^+^. All experiments on animals were performed under UK Home Office authorization.

### Flow cytometry

All BM and FL samples were stained and analyzed as described previously (Kranc et al., 2009; Mortensen et al., 2011; Guitart et al., 2013; Vukovic et al., 2016). BM cells were obtained by crushing tibias and femurs with a pestle and mortar. FL cells were obtained by mashing the tissue through a 70-µm strainer. Single-cell suspensions from BM, FL, or PB were incubated with Fc block and then stained with antibodies. For HSC analyses, after incubation with Fc block, unfractionated FL or BM cell suspensions were stained with lineage markers containing biotin-conjugated anti-CD4, anti-CD5, anti-CD8a, anti-CD11b (not used in FL analyses), anti-B220, anti–Gr-1, and anti-Ter119 antibodies together with APC-conjugated anti–c-Kit, APC/Cy7-conjugated anti–Sca-1, PE-conjugated anti-CD48, and PE-Cy7–conjugated anti-CD150 antibodies. Then, biotin-conjugated antibodies were stained with Pacific blue–conjugated or PerCP-conjugated streptavidin. To distinguish CD45.2^+^ donor–derived HSCs in recipient mice, FITC-conjugated anti-CD45.1 and Pacific blue–conjugated anti-CD45.2 antibodies were included in the antibody cocktail. The multilineage reconstitution of recipient mice was determined by staining the BM or PB cell suspensions of the recipient mice with FITC-conjugated anti-CD45.1, Pacific blue–conjugated anti-CD45.2, PE-conjugated anti-CD4 and -CD8a, PE/Cy7-conjugated anti–Gr-1, APC-conjugated anti-CD11b, APC-Cy7–conjugated anti-CD19, and anti-B220. In all analyses, 7-AAD or DAPI was used for dead cell exclusion. Flow cytometry analyses were performed using an LSRFortessa flow cytometer (BD). Cell sorting was performed on a FACSAria Fusion cell sorter (BD).

### CFC assays

CFC assays were performed using MethoCult (M3434; STEMCELL Technologies). Two replicates were used per group in each experiment. Colonies were tallied at day 10.

### Leukemic transformation

LSK cells were sorted from FLs of 14.5-dpc embryos after c-Kit (CD117) enrichment using magnetic-activated cell-sorting columns (Miltenyi Biotec). 10,000 LSK cells were simultaneously transduced with mouse stem cell virus (MSCV)–*Meis1a-puro* and MSCV–*Hoxa9-neo* retroviruses and subsequently subjected to three rounds of CFC assays in MethoCult (M3231) supplemented with 20 ng/ml stem cell factor, 10 ng/ml IL-3, 10 ng/ml IL-6, and 10 ng/ml granulocyte/macrophage stem cell factor. Colonies were counted 6–7 d after plating, and 2,500 cells were replated. Similarly, 200,000 FL c-Kit^+^ cells were transduced with MSCV–*AML1-ETO9a-neo*, MSCV–*MLL-AF9-neo*, or MSCV–*MLL-ENL-neo* and subsequently plated into methylcellulose.

### Transplantation assays

Lethal irradiation of CD45.1^+^/CD45.2^+^ recipient mice was achieved using a split dose of 11 Gy (two doses of 5.5 Gy administered at least 4 h apart) at a mean rate of 0.58 Gy/min using a Cesium 137 irradiator (GammaCell 40; Best Theratronics). For sublethal irradiation, the recipient mice received a split dose of 7 Gy (two doses of 3.5 Gy at least 4 h apart).

For primary transplantations, 100 HSCs (LSKCD48^−^CD150^+^CD45.2^+^) sorted from FLs of 14.5-dpc embryos or 200,000 unfractionated FL cells were mixed with 200,000 support CD45.1^+^ BM cells and injected into lethally irradiated (11 Gy delivered in a split dose) CD45.1^+^/CD45.2^+^ recipient mice. For secondary transplantations, 2,000 CD45.2^+^ LSK cells sorted from BM of primary recipients were mixed with 200,000 support CD45.1^+^ wild-type BM cells and retransplanted. For adult BM transplantations, 500,000 CD45.2^+^ BM cells were mixed with 500,000 support CD45.1^+^ wild-type BM cells and injected into lethally irradiated CD45.1^+^/CD45.2^+^ recipient mice. All recipient mice were analyzed 18–20 wk after transplantation, unless otherwise stated.

For leukemia induction, 100,000 *Meis-1*/*Hoxa9*-transduced c-Kit^+^ cells were transplanted into CD45.1^+^/CD45.2^+^ sublethally irradiated (7 Gy delivered in a split dose) recipient mice. The mice were monitored for AML development. For secondary transplantation, 10,000 LICs (CD45.2^+^c-Kit^+^ cells) were sorted from BM of primary recipients and transplanted into secondary CD45.1^+^/CD45.2^+^ sublethally irradiated recipient mice.

### Inducible *Mx1-Cre*–mediated gene deletion

Mice were injected intraperitoneally six to eight times every alternate day with 300 µg pIpC (GE Healthcare) as previously described (Kranc et al., 2009; Guitart et al., 2013).

### Administration of NAC

Pregnant females received 30 mg/ml of NAC (Sigma-Aldrich) in drinking water (pH was adjusted to 7.2–7.4 with NaOH). For transplantation experiments, CD45.1^+^/CD45.2^+^ recipient mice were treated with 30 mg/ml NAC in drinking water 7 d before irradiation and remained under NAC treatment for the duration of the experiment. The water bottle containing NAC was changed twice per week.

### Oxygen consumption assays

OCR measurements were made using a Seahorse XF-24 analyzer (Seahorse Bioscience) and the XF Cell Mito Stress Test kit as previously described (Wang et al., 2014). In brief, c-Kit^+^ cells from FLs of 14.5-dpc embryos were plated in XF-24 microplates precoated with cell-tak (BD) at 250,000 cells per well in XF Base medium supplemented with 2 mM pyruvate and 10 mM glucose, pH 7.4. OCR was measured three times every 6 min for basal value and after each sequential addition of oligomycin (1 µM), FCCP (1 µM), and finally concomitant rotenone and antimycin A (1 µM). Oxygen consumption measurements were normalized to cell counts performed before and after each assay.

### Metabolite detection by liquid chromatography–mass spectrometry (LC-MS)

Metabolites from c-Kit^+^ cells from FLs of 14.5-dpc embryos were extracted into 50% methanol/30% acetonitrile and measured as previously described (Adam et al., 2013).

### Western blotting

Protein extracted from FL c-Kit^+^ cells of 14.5-dpc embryos was subjected to a 10% SDS–PAGE and then transferred onto a polyvinylidene fluoride membrane and immunoblotted with anti-Fh1, anti–2-SC, and anti–Hif-1α as previously described (Adam et al., 2011; Bardella et al., 2012). Anti-H3K4me3 (07-473; EMD Millipore), anti-H3K9me3 (ab8898; Abcam), anti-H3K27me3 (07-449; EMD Millipore), and anti-H3K36me3 (ab9050; Abcam) were used to determine levels of trimethylated H3. Anti-actin (A5316; Sigma-Aldrich), anti-tubulin (2146S; Cell Signaling Technology), and anti-H3 (ab1791; Abcam) immunoblots were used as loading controls.

### RT–quantitative PCR

Gene expression analyses were performed as described previously (Kranc et al., 2009; Mortensen et al., 2011; Guitart et al., 2013). Differences in input cDNA were normalized with *Actb* (β-actin) expression.

### ATP production

10,000 c-Kit^+^ cells from FLs of 14.5-dpc embryos were cultured in DMEM supplemented with either 25 mM glucose or 25 mM galactose. At 0 and 24 h after incubation, the cells were lysed, and ATP content was measured by luminescence using CellTiterGlo Assay (Promega).

### Measurement of mitochondrial membrane potential

50,000 FL c-Kit^+^ cells from 14.5-dpc embryos were incubated for 15 min at 37°C in 25 nM tetramethylrhodamine methyl ester (T-668; Thermo Fisher Scientific) and analyzed using the LSRFortessa flow cytometer.

### Gene expression profiling and bioinformatics analyses

RNA from sorted FL Lin^−^c-Kit^+^ cells was isolated by standard phenol/chloroform extraction. cDNA was synthesized from 50 ng of total RNA using the Ambion WT Expression kit (Thermo Fisher Scientific). Labeled, fragmented cDNA (GeneChip WT Terminal Labeling and Controls kit; Affymetrix) was hybridized to Mouse Gene 2.0 arrays for 16 h at 45°C and 60 rpm (GeneChip Hybridization, Wash, and Stain kit; Affymetrix). Arrays were washed and stained using the Fluidics Station 450 (Affymetrix) and scanned using a GeneArray Scanner (3000 7G; Hewlett-Packard). The microarray gene expression data have been deposited in the ArrayExpress database under accession no. E-MTAB-5425.

For bioinformatics analyses, a total of seven arrays (*n* = 3 *Fh1^fl/fl^*; *n* = 4 *Fh1^fl/fl^;Vav-iCre*) were quality control analyzed using the arrayQualityMetrics package in Bioconductor. Normalization of the 29,638 features across all arrays was achieved using the robust multiarray average expression measure. Pairwise group comparisons were undertaken using linear modeling (LIMMA package in Bioconductor). Subsequently, empirical Bayesian analysis was applied, including vertical (within a given comparison) p-value adjustment for multiple testing, which controls for false discovery rate.

### Gene set enrichment analysis (GSEA)

Gene expression differences were ranked by difference of log expression values, and this ranking was used to perform GSEA (Subramanian et al., 2005) on gene lists in the Molecular Signatures Database (MSigDB; version 5.2). The following datasets were used for analyses presented in Fig. 4: (a) hallmark, unfolded protein response; (b) gene ontology, intrinsic apoptotic signaling pathway in response to endoplasmic reticulum stress; (c) reactome, translation; and (d) NFE2L2.V2.

### shRNA-mediated *FH* knockdown

THP-1 cells were transduced with lentiviruses expressing shRNAs (shRNA1, 5′-TAATCCTGGTTTACTTCAGCG-3′ [TRCN0000052463]; shRNA2, 5′-AAGGTATCATATTCTATCCGG-3′ [TRCN0000052464]; shRNA3, 5′-TTTATTAACATGATCGTTGGG-3′ [TRCN0000052465]; and shRNA Scr, 5′-TTCTCCGAACGTGTCACGTT-3′; RNAi Consortium; GE Healthcare). Transduced THP-1 cells were grown in the presence of 5 µg/ml puromycin.

### ROS analysis

c-Kit^+^ cells were stained with 2.5 nM CellROX (C10491; Thermo Fisher Scientific) based on the manufacturer’s protocol and analyzed by FACS.

### Statistical analyses

Statistical analyses were performed using Prism 6 (GraphPad Software). P-values were calculated using a two-tailed Mann-Whitney *U* test unless stated otherwise. Kaplan-Meier survival curve statistics were determined using the log-rank (Mantel-Cox) test.

### Online supplemental material

Table S1 shows neutropenia in patients with recessive *FH* mutations. Table S2 shows hematopoiesis-specific *Fh1* deletion results in embryonic lethality. Table S3 shows *FH^Cyt^* rescues embryonic lethality in *Fh1^fl/fl^;Vav-iCre* mice.
